# Antitumor Effect of Poplar Propolis on Human Cutaneous Squamous Cell Carcinoma A431 Cells

**DOI:** 10.3390/ijms242316753

**Published:** 2023-11-25

**Authors:** Chuang Zhang, Yuanyuan Tian, Ao Yang, Weihua Tan, Xiaoqing Liu, Wenchao Yang

**Affiliations:** 1 College of Animal Science (College of Bee Science), Fujian Agriculture and Forestry University, Fuzhou 350002, China; chuangzhang12@163.com (C.Z.); tianyuan4601@126.com (Y.T.); ya104110@126.com (A.Y.); lxq7597@163.com (X.L.); 2 College of Juncao Science and Ecology, Fujian Agriculture and Forestry University, Fuzhou 350002, China; 3 College of Food Science, Fujian Agriculture and Forestry University, Fuzhou 350002, China; tanweihuayes@126.com

**Keywords:** propolis, human cutaneous squamous cell carcinoma, A431 cell line, proteomics, xenograft tumor nude mice

## Abstract

Propolis is a gelatinous substance processed by western worker bees from the resin of plant buds and mixed with the secretions of the maxillary glands and beeswax. Propolis has extensive biological activities and antitumor effects. There have been few reports about the antitumor effect of propolis against human cutaneous squamous cell carcinoma (CSCC) A431 cells and its potential mechanism. CCK-8 assays, label-free proteomics, RT–PCR, and a xenograft tumor model were employed to explore this possibility. The results showed that the inhibition rate of A431 cell proliferation by the ethanol extract of propolis (EEP) was dose-dependent, with an IC_50_ of 39.17 μg/mL. There were 193 differentially expressed proteins in the EEP group compared with the control group (*p* < 0.05), of which 103 proteins (53.37%) were upregulated, and 90 proteins (46.63%) were downregulated. The main three activated and suppressed Kyoto Encyclopedia of Genes and Genomes (KEGG) pathways were extracellular matrix (ECM)-receptor interaction, amoebiasis, cell adhesion molecules (CAMs), nonalcoholic fatty liver disease (NAFLD), retrograde endocannabinoid signaling, and Alzheimer’s disease. The tumor volume of the 100 mg/kg EEP group was significantly different from that of the control group (*p* < 0.05). These results provide a theoretical basis for the potential treatment of human CSCC A431 cell tumors using propolis.

## 1. Introduction

Cutaneous squamous cell carcinoma (CSCC) is a malignant tumor originating from keratinocytes of the epidermis or appendages and the primary cause of death among nonmelanoma skin tumors [1]. In recent years, approximately 15–35 people in every 100,000 people have been diagnosed with CSCC, with an increase of 2–4% every year, which has become a worldwide threat to public health [2,3]. CSCC is mostly diagnosed in elderly patients, and the ratio of men to women is approximately 2~3:1. Most CSCC occurs in body parts, such as the head and face, exposed to ultraviolet light [4]. Currently, surgery, radiotherapy, and chemotherapy are the main treatment strategies. Surgery is an effective way to remove tumors, but it is not easy to detect cancer at an early stage. Chemotherapy and radiography treatments have side effects, such as damaging normal cells and drug resistance, which reduce the quality of life of patients. The search for alternative treatments is urgently needed.

Propolis (bee glue) is a kind of viscous substance processed by Western honeybee workers that collect mucilage, gums, resins, and lattice from plants, such as *Pinus* spp., *Prunus* spp., *Acacia* spp., *Betula pendula*, *Aesculus hippocastanum*, *Salix alba*, *Baccharis dracunculifolia*, and *Dalbergia ecastaphyllum* (L.) Taub., and the collections are mixed with the secretion of worker maxillary glands and beeswax [5,6]. The chemical composition of propolis, mainly including flavonoids, flavonols, flavanones, dihydroflavonoids, and phenylpropane derivatives, varies depending on the plant, geographical origin, and harvest season [7]. There are significant differences in color, active components, and harvesting tools of poplar propolis, red and green propolis [6]. Green Brazilian propolis is derived mainly from the leaf resin of *Baccharis dracunculifolia*, and red propolis is from the resin of *Dalbergia ecastophyllum*, while poplar propolis (brown color) is from the resin of *Populus nigra* L. [6]. The main active components of green propolis were derivatives of phenylpropanoids and diterpenes, chlorophyll, and small amounts of flavonoids, and those of red and poplar propolis were flavonoids [7]. Propolis has a wide range of biological activities, such as antibacterial, antifungal, antiviral, antiparasitic, antioxidant, antitumor, anti-inflammatory, anti-ulcer, and antidiabetic effects [5,8].

The antitumor activity of propolis has attracted much attention. In recent years, the antitumor effects of propolis on cancer and the relevant mechanisms have been reported in in vitro studies on colorectal, lung, breast, melanoma, gastric, lymphoma, tongue, and skin [7,8,9,10,11,12,13,14,15]. Many active ingredients in propolis, such as flavonoids and caffeic acid phenylethyl ester, inhibit tumor activity. Among them, caffeic acid phenylethyl ester has a specific and targeted killing effect on docetaxel-resistant prostate cancer cells [10]. Flavonoids block the cell cycle and then inhibit the proliferation of a human breast cancer cell line (MCF-7) [11]. There are selective antitumor effects of propolis on normal gingival fibroblasts and tongue cancer cells [12]. Red propolis has a cytotoxic effect on the breast cancer MDA MB-231 cell line, which is related to the PI3K/Akt and ERK1/2 pathways [13]. Propolis reduces mitochondrial membrane potential and changes the expressions of specific tumor suppressors (miR-34, miR-15a, and miR-16-5p) and carcinogenic (miR-21) miRNAs by increasing the levels of proapoptotic proteins (p21, Bax, p53, p53 Ser46, and p53 Ser15) in the MCF-7 cell line [14]. Brazilian propolis reduces the proliferation of a head and neck squamous cell carcinoma (HNSCC) cell line [15]. The antitumor effect of poplar propolis on CSCC A431 cells and its potential mechanism are unclear.

In this study, Cell Counting Kit-8 (CCK-8) assays, label-free proteomics, RT–PCR, and a xenograft tumor nude mouse model were employed to determine the effect of propolis on the proliferation, differentially expressed proteins, related pathways in A431 cells, and tumor volume in an animal xenograft tumor model.

## 2. Results

### 2.1. Components of Ethanol Extract of Propolis 

**Figure 1:**
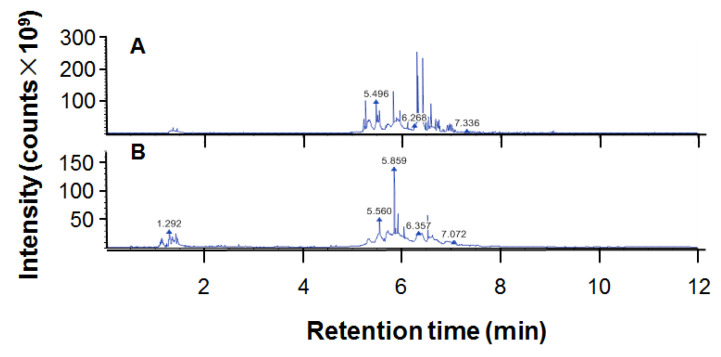
The chromatogram of ethanol extract of propolis (EEP) for UPHLC-MS/MS: (**A**) negative polarity mode; (**B**) positive polarity mode.

The total flavonoid content of ethanol extract of propolis (EEP) was 32.04 ± 0.89/100 g. The chromatogram for UPHLC-MS/MS of propolis is shown in Figure 1. The 214 chemical components of EEP dissolved in methanol are presented in Table 1.

### 2.2. The Antitumor Effect of Ethanol Extract of Propolis (EEP)

**Figure 2:**
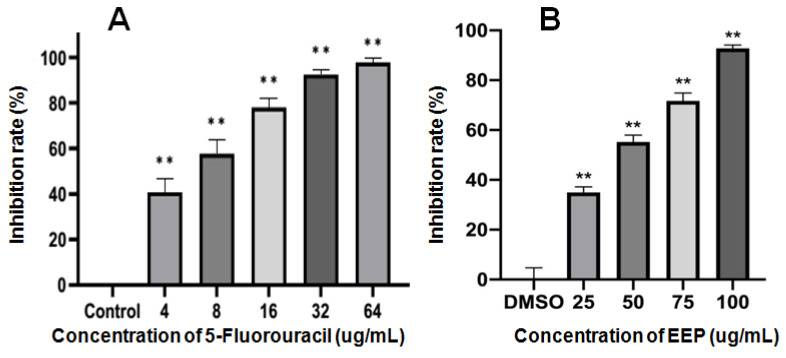
Inhibitory rates of 5-Fluorouracil (**A**) and ethanol extract of propolis (EEP) (**B**) against the proliferation of A431 cells for 48 h (** indicates significant differences compared with the solvent control group. *p* < 0.01).

There were no significant differences between the viability of A431 cells in the solvent control group and the blank control group. EEP inhibited the proliferation of A431 cells. DMSO (0.05%) had no effect on A431 cells. The inhibition rates are shown in Figure 2. The IC_50_ of 5-FU and EEP against A431 cells for 48 h was 6.57 and 39.17 µg/mL, respectively.

**Figure 3:**
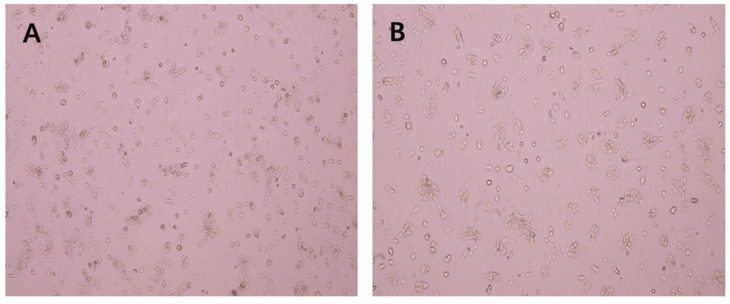
Effect of ethanol extract of propolis (EEP) on the morphology of A431 cells (100×): (**A**): Control, (**B**) IC_50_ EEP group.

The morphology of A431 cells after EEP treatment for 48 h is shown in Figure 3. The growth of the cells in the control group was normal, and the treated cells were irregular in shape and distributed in sheets. The number of cells treated with EEP in the microscope field of view decreased, and the cell adhesion ability was weakened with some floating cells.

### 2.3. The Differentially Expressed Proteins

There were 103 upregulated and 90 downregulated differentially expressed proteins (DEPs) between the EEP group and the control group (screened by FC > 2.0 or FC < 0.5 and *p* < 0.05). Partial DEPs (*p* < 0.01) are shown in Table 2.

**Figure 4:**
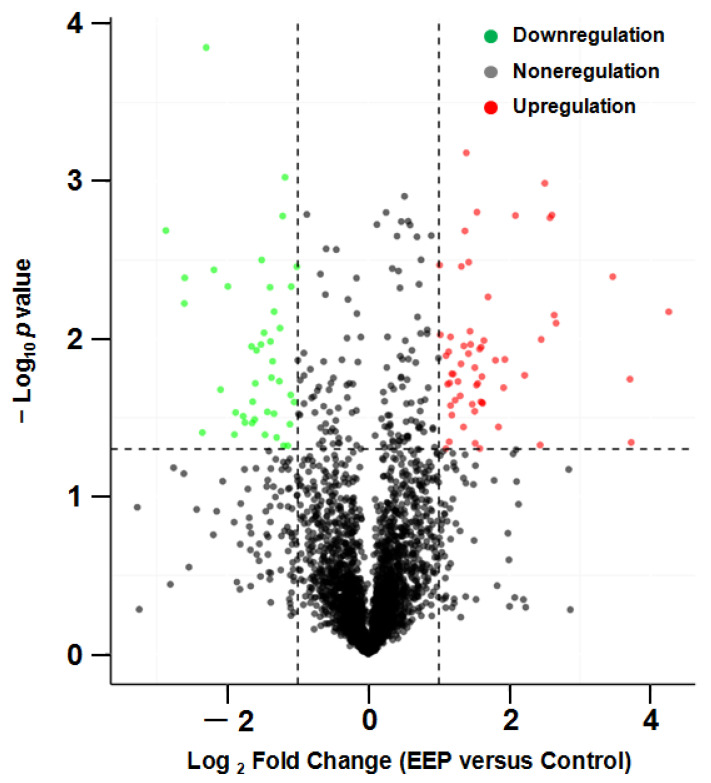
Volcano plot of proteins in A431 cells treated with ethanol extract of propolis (EEP) versus control cells.

**Figure 5:**
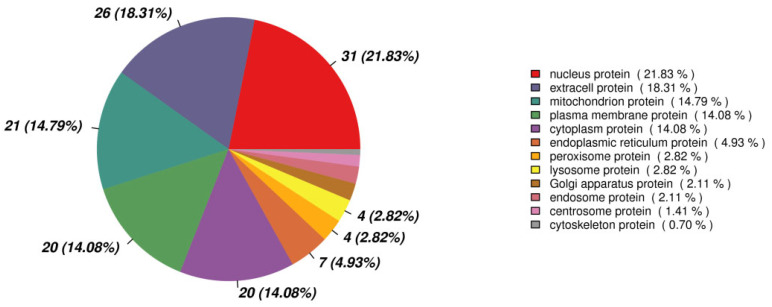
The subcellular localization of the differentially expressed proteins.

**Figure 6:**
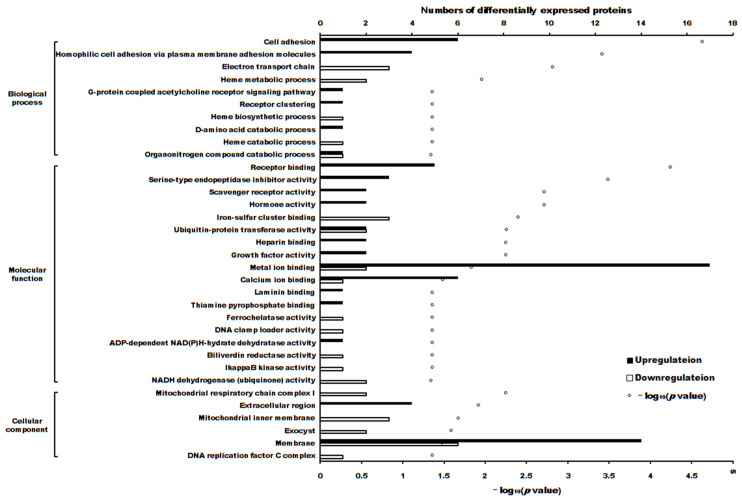
The differentially expressed proteins enriched in Gene Ontology terms (*p* < 0.05).

**Figure 7:**
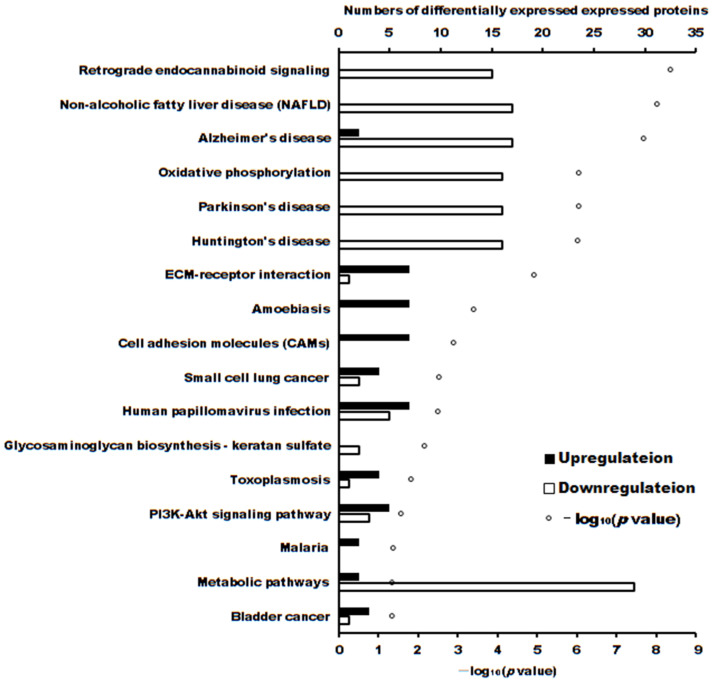
The number of differentially expressed proteins enriched in Kyoto Encyclopedia of Genes and Genomes (*p* < 0.05).

The volcano plot of proteins in the two groups is shown in Figure 4. The subcellular localization of the DEPs is shown in Figure 5. The DEPs subjected to Gene Ontology (*p* < 0.05) and Kyoto Encyclopedia of Genes and Genomes (*p* < 0.05) analyses are shown in Figure 6 and Figure 7, respectively.

These DEPs played roles in different pathways. The significantly enriched pathways (*p* < 0.05) of upregulated and downregulated proteins were separately analyzed, as shown in Table 3.

**Figure 8:**
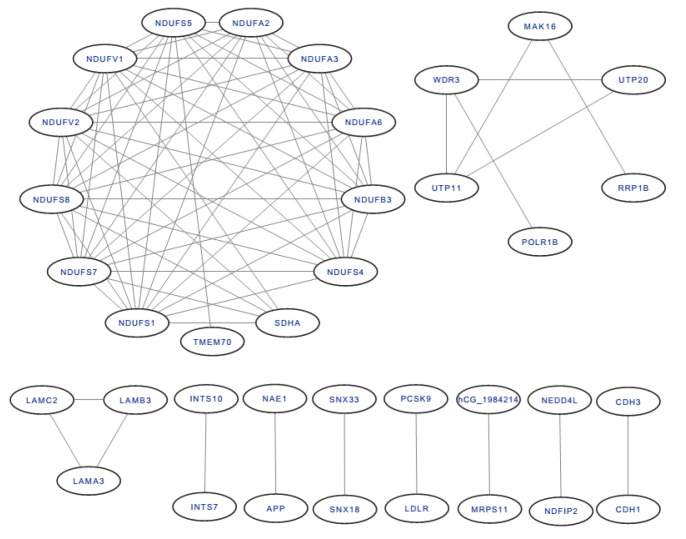
The protein-protein interaction of DEPs.

All of the DEP interactions are shown in Figure 8. Among the interacting proteins, NADH dehydrogenase [ubiquinone] flavoprotein 2 (mitochondrial), mitochondrial NADH-ubiquinone oxidoreductase 75 kDa subunit, NADH dehydrogenase [ubiquinone] flavoprotein 1 (mitochondrial), NADH dehydrogenase (ubiquinone) Fe–S protein 5, 15 kDa (NADH-coenzyme Q reductase), NADH dehydrogenase [ubiquinone] iron–sulfur protein 8 (mitochondrial), and NADH dehydrogenase [ubiquinone] iron–sulfur protein 7 (mitochondrial) had the most protein interactions, with 11 DEPs.

### 2.4. Relative Gene Expression

**Figure 9:**
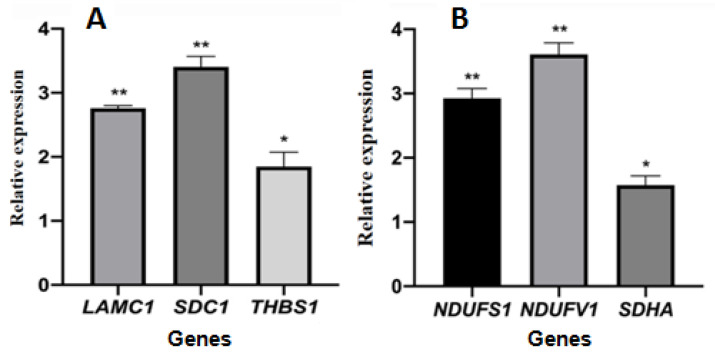
The relative gene expression of selected genes encoding differentially expressed proteins. (**A**): genes coded proteins enriched in ECM-receptor interaction pathway; (**B**): genes coded proteins enriched in oxidative phosphorylation pathway. The symbols * and ** indicates significant differences compared with the solvent control group, *p* < 0.05 or *p* < 0.01, respectively.

The cycle threshold (ct) data of selected genes is shown in Supplementary Table S1. The relative gene expression levels of selected genes encoding differentially expressed proteins are shown in Figure 9. The expression levels of the three genes *LAMC1*, *SDC1*, and *THBS1* involved in the ECM-receptor interaction pathway were significantly upregulated in the treated group compared with the control group, and the gene expression was consistent with the expression of proteins. The expression levels of three genes, *NDUFS1*, *NDUFV1*, and *SDHA*, involved in the oxidative phosphorylation pathway, were significantly upregulated and inconsistent with the expression of proteins.

### 2.5. The Effect of EEP on A431 Cell Xenograft Tumors in Nude Mice

The tumor volumes of nude mice in the control group, solvent group, 50 mg/kg propolis group, and 100 mg/kg propolis group after 12 days of gavage are shown in Table 4. There was a significant difference in the 100 mg/kg propolis group compared with the control group (*p* < 0.05), which indicated that the 100 mg/kg propolis group had in vivo inhibitory effects on A431 cell tumors.

**Figure 10:**
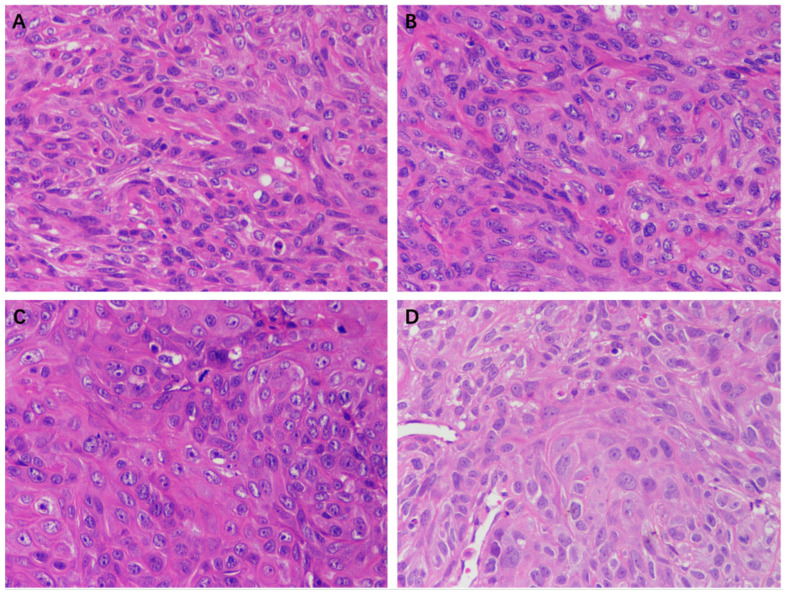
HE staining of tumors in nude mice of different groups (400×). (**A**) Control group, (**B**) Solvent control group, (**C**) 50 mg/kg EEP group, and (**D**) 100 mg/kg EEP.

The HE staining results of the tumor tissue of the EEP, solvent control, and control groups are shown in Figure 10. A large number of tumor cells were observed in each group except in the 100 mg/kg EEP group. The morphology and size of cells varied and exhibited atypia, with enlarged nucleoli and unclear cell spacing. There was a small amount of cell necrosis in the control group, solvent control group, and 50 mg/kg group, while a large amount of cell necrosis was observed in the 100 mg/kg EEP group.

## 3. Discussion

Propolis exhibits antitumor activity against different cell lines. Brazilian red propolis (from Brejo Grande, Brazil) can inhibit the growth of cancer cells, and after 24 h of treatment, the IC_50_ values against Hep-2 cells and HeLa cells were 63.48 ± 3.30 μg/mL and 81.40 ± 6.40 μg/mL, respectively [16]. EEP (from Ardabil, Iran) has dose-dependent toxic effects on both KB and A431 cancer cells. The IC_50_ values of EEP in the KB cell line and A431 cell line were 40 ± 8.9 μg/mL and 98 μg/mL, respectively, after 48 h of incubation [17]. The IC_50_ values of EEPs (from Podlasie, Masovia, and West Pomerania; Poland) against tongue cancer cells treated for 24 h were approximately 88 µg/mL, 110 µg/mL, and 150 µg/mL, respectively [12]. The IC_50_ values of EEPs range from 26.33 to 143.09 μg/mL against the human colon cancer cell line HCT-16 [18]. The IC_50_ values of EEP (from Phayao, Chiang Mai, and Nan, Thailand) against A549 cells were 106 ± 0.004 µg/mL, 199 ± 0.009 µg/mL, and 87 ± 0.012 µg/mL, respectively, and for HeLa cells were 81 ± 0.006 µg/mL, 116 ± 0.023 µg, and 54 ± 0.005 µg/mL, respectively [19]. The IC_50_ of EEP (from Hebei Province, China) against the 5 × l0^5^/mL DLBCL SU-DHL-2 cell line for 24 h was 5.729 μg/mL [9]. In this study, the IC_50_ of EEP (same as [9]) against A431 cells for 48 h was 39.17 μg/mL (Figure 3). These different median lethal doses against tumor cell lines may be related to the type of cancer cells, concentration of cancer cells, incubation duration, botanical origin of propolis, extraction process of propolis, and storage of propolis.

The cytotoxicity mechanism of propolis against A431 tumor cells was different. Proteins play important roles in the proliferation of cells. Label-free proteomics is commonly used to explore DEPs in cells subjected to different treatments [20,21,22]. In this manuscript, there were 103 upregulated and 90 downregulated DEPs between treatment and control cells. GO enrichment and KEGG enrichment analysis (as shown in Figure 6 and Figure 7) showed that the main upregulated proteins enriched in the ECM-receptor interaction and cell adhesion molecule (CAM) pathways inhibits the ability of A431 cells to metastasize and invade. The downregulated proteins were mainly enriched in adenosine triphosphate (ATP) production by downregulating the main proteins of the retrograde endocannabinoid signaling and oxidative phosphorylation pathways, thereby reducing adenosine triphosphate (ATP) production and inhibiting the proliferation of A431 cells.

The most significantly enriched pathway of the upregulated DEPs was the ECM-receptor interaction pathway. The interaction between tumor cells and extracellular matrix (ECM) components, such as laminin, fibronectin, and collagen, plays a crucial role in tumor invasion and metastasis. The genes *LAMC1*, *SDC1*, and *THBS1*, which are involved in the ECM-receptor interaction pathway, were upregulated. Similar results were also found in gastric cancer, in which the upregulated genes were *COL1A2* and *COL6A3* [23] or *COL6A3*, *COL3A1*, and *COL1A1* [24]. There were seven upregulated proteins involved in the ECM-receptor interaction pathway, which is associated with an enhanced migratory/invasive capacity of ovarian cancer cells [25] and non-small cell lung cancer tumors [26,27]. Most of the upregulated DEPs, except Thrombospondin 1, Agrin, and Syndecan-1, involved in the ECM-receptor interaction pathway were also enriched in the amoebiasis pathway. Differentially expressed genes related to cervical cancer were enriched in amoebiasis and other pathways [28,29]. Similar differentially expressed genes were also found in colorectal cancer cells with cetuximab insensitivity [30]. The differentially expressed proteins were involved in amoebiasis pathways of non-small cell lung cancer [26] and gastric cancer tumors of the patients [27].

Another important pathway of the upregulated DEPs was the amoebiasis pathway. The amoebiasis pathway was significantly enriched and identified as one of the important processes or signaling pathways of melanoma metastasis [31,32], the pathogenesis of nasopharyngeal carcinoma [33], carcinogenesis and pathogenesis of cervical cancer [28], breast cancer [34], and gastric cancer [35] by bioinformatics analysis based on the Gene Expression Omnibus database. Our result is in accordance with these previous scientific studies.

The third pathway of the upregulated DEPs was the cell adhesion molecule (CAM) pathway. CAMs, having four main groups including cadherins, integrins, selectins, and immunoglobulins, are primarily glycoproteins on cell surface membranes and can promote homeostasis between cells and between cells and the extracellular matrix. With higher levels of neural cell adhesion molecule expression, neuroblastoma cells have more intense homophilic tumor binding [36]. Knockdown of E-cadherin and cell adhesion molecule 1-related genes decreased cell growth, migration, and cell-to-cell adhesion of BAP1-mutant uveal melanoma cells [37]. High expression of prostaglandin F2 receptor inhibitor (PTGFRN), a type I (single pass) transmembrane Ig superfamily of CAM, could protect cells from apoptosis, thereby promoting growth and migration in glioblastoma cells [38]. It was also shown that the CAM pathway is one of the key processes or signaling pathways of melanoma metastasis and the pathogenesis of nasopharyngeal carcinoma [31,32,33]. CAMs may be the stress response to adverse factors on cancer cells.

The most significantly downregulated pathway was the nonalcoholic fatty liver disease (NAFLD) pathway. NAFLD affected the cell cycle and p53 pathways. SNORA71A knockdown in HT-29 cells led to significant inhibition of cell migration and invasion ability, which targeted LBP to participate in NAFLD in colorectal cancer cells [39]. Corosolic acid inhibited NAFLD-related hepatocellular carcinoma progression by downregulating the NAFLD pathway [40]. Levodopa downregulates oxidative phosphorylation (OXPHO), NAFLD, and Parkinson’s disease-related pathways to inhibit esophageal squamous cell carcinoma cells [41]. Similar results were also obtained in this experiment, in which the OXPHO and Parkinson’s disease-related pathways were also suppressed. OXPHO mainly occurs in the inner mitochondrial membrane of eukaryotic cells or the cytoplasm of prokaryotic organisms [42]. Many tumors require energy from the mitochondria for biosynthesis to synthesize ATP through OXPHO [43]. The average contribution of OXPHOS to ATP generation in normal cells is 80% and 83% in cancer cells [44]. Triple-negative breast cancer (TNBC) cells can be inhibited by a decrease in OXPHOS caused by OXPHOS inhibitors, which causes ATP deficiency that cannot be fully compensated by other mechanisms [45]. Oxidative phosphorylation also acts on other cancer cells, such as liver cancer [46], rectal cancer [47], and pancreatic cancer cells [48]. OXPHO is now widely used as a therapeutic target in cancer [49].

Another suppressed pathway was retrograde endocannabinoid signaling. This pathway was also suppressed in retinoblastoma [50], glioblastoma [51], and glioma patients [52]. The metabolites were also enriched in the retrograde endocannabinoid signaling pathway in breast cancer cells treated with *Faecalibacterium prausnitzii* [53]. This pathway was also mainly enriched in DEPs and DEGs related to nonfunctional pituitary adenoma [54]. The DEPs between the tumor and adjacent healthy tissue of patients with diffuse gastric cancer and those of patients with advanced gastric cancer were enriched in this pathway [55]. Other suppressed pathways were Alzheimer’s disease-related pathways, Huntington’s disease-related pathways, metabolic pathways, and glycosaminoglycan biosynthesis—keratan sulfate. The proteins involved in these pathways were downregulated and inhibited the growth of A431 cells.

The expression levels of genes subjected to RT–PCR were not completely consistent with the protein expression levels. Some studies have pointed out that transcription levels alone are not sufficient to predict protein levels in many cases [56,57] because the protein concentration is affected by both transcription and translation. Cancer-related genetic changes can affect proteins involved in nearly all levels of transcriptional control [58].

This experiment showed that EEP inhibited tumor growth in nude mice. It was also found that the growth of gastric pyloric tumors and colorectal cancer was inhibited by a diet containing propolis [59,60]. Propolis has immunological enhancement activity [61], which can enhance the immune system of mice and then nonspecificly inhibit tumor growth.

The limitation of this study is that metabonomics, cancer stem cell, molecular docking, or other methods can be employed to explore more accurately the regulation of the EPP antitumor approach against the A431 cells for new drug development. More research about the cell cycle of A431 cells inhibited by EEP and the active components, such as caffeic acid, dihydro cinnamic acid, and p-coumaric acid, or others, who play the main antitumor effect can be determined in the future.

## 4. Materials and Methods

### 4.1. Propolis Samples and Its Chemical Components Determination

The crude poplar propolis sample and extraction procedure of ethanol extract of propolis (EEP) were the same as in our previous report [9].

The total flavonoid content determination of EEP was performed using the spectrophotometer method according to the national standards for propolis in China (GB/T 24283-2018)[62]. Rutin was used as a standard substance to determine the absorbance of the samples at 510 nm.

The chemical components of EEP were determined using a UHPLC-MS/MS system (Vanquish UHPLC system (Thermo Fisher Scientific Inc., Germering, Germany) coupled with an Orbitrap Q ExactiveTMHF-X mass spectrometer (ThermoFisher, Germering, Germany)) by Untargeted Metabolomics mothed and performed by Novogene Co., Ltd. (Beijing, China). Simply, The EEP samples (1 mL) were resuspended with prechilled 80% methanol by well vortex and then incubated on ice for 5 min and centrifuged at 15,000× *g*, 4 °C for 15 min. The supernatant was diluted to 53% methanol final concentration by LC-MS grade water. The solution was transferred to an Eppendorf tube and subsequently centrifuged at 15,000× *g*, 4 °C for 15 min. The elution conditions and processes were the same as in reference [63]. Q ExactiveTM HF-X mass spectrometer was operated in positive/negative polarity mode with a spray voltage of 3.5 kV, capillary temperature of 320 °C, sheath gas flow rate of 35 psi, and aux gas flow rate of 10 L/min, S-lens RF level of 60, Aux gas heater temperature of 350 °C.

### 4.2. Antitumor Bioassay

The human skin squamous cell carcinoma A431 cell line (purchased from Wuhan Purosai Life Sciences Co., Ltd., Wuhan, China) was cultured in a special complete culture medium (Wuhan Purosai Life Sciences Co., Ltd.) in a 5% CO_2_ humidified incubator at 37 °C (C150, Binder, Tuttlingen, German).

EEP (0.1 g) was dissolved in 0.5 mL DMSO and diluted with a complete culture medium at a concentration of 1 mg/mL. Propolis solution was diluted with a complete medium to 100, 75, 50, and 25 µg/mL. DMSO (0.05%, *v/v*; equal to DMSO in the 100 µg/mL propolis group) was added to the complete medium as a solvent control.

The A431 cells were irrigated with PBS buffer (pH 7.2–7.4, Sinopharm Chemical Reagent Co., Ltd., Shanghai, China) twice and then digested with 1 mL 0.25% trypsin solution (HyClone, Thermo Scientific, Waltham, UT, USA). The digested solution was centrifuged at 137× *g* for 5 min. The sediment was suspended in 2 mL of complete culture medium. The concentration was determined via 0.4% trypan blue staining (Beijing Solarbio Science & Technology Co., Ltd., Beijing, China). At a beginning concentration of 5 × 10^4^ cells/mL, 100 µL cell suspension was added in 96-well plates with 6 repetitions per treatment. After 48 h of cell culture with EEP 100, 75, 50, and 25 µg/mL, and 0.05% DMSO, the viability of cells was determined by a CCK8 kit (DOJINDO, Kumamoto, Japan) at 450 nm using a microplate reader (1510, Thermo Fisher Waltham, MA, USA). The IC_50_ of EEP on A431 cells for 48 h was calculated using GraphPad Prism 8.4.3 for Windows (GraphPad Software, Inc., La Jolla, CA, USA). 5-Fluorouracil (HPLC grade; purchased from Sigma-Aldrich Co., St. Louis, MO, USA) at 0, 4, 8, 16, 32, and 64 µg/mL were employed to determine the IC_50_ value against A431 cells.

A431 cells in the logarithmic growth phase were digested with trypsin and seeded in 6-well plates at 1.5 × 10^5^ cells/well. After 24 h of cell culture, the cells were treated with control, and IC_50_ EEP solution for 48 h, and then the morphological changes of the cells were observed using an inverted microscope (TS-100f, Nikon, Tokyo, Japan).

### 4.3. Differentially Expressed Proteins in A431 Cells Treated with Propolis

The A431 cells were treated as described for the morphology observation experiment, which was also treated with control and IC_50_ EEP solution. After these cells were treated with propolis or non-propolis for 48 h, the culture medium was removed. Then, the cells were irrigated twice with precooled PBS buffer, digested, and irrigated twice again. Cells were collected in a centrifuge tube (1.5 mL) after centrifugation. Therefore, these cells were frozen in liquid nitrogen and further stored in a refrigerator at −80 °C (Haier Biomedical, Qingdao, China).

The extraction and concentration determination of total proteins and spectra of proteins were performed as described in our previous report [9].

### 4.4. Detection of Relative Gene Expression

According to the proteomics results, the genes coded NDUFS1, NDUFV1, and SDHA proteins involved in oxidative phosphorylation and LAMC1, SDC1, and THBS1 ECM-receptor interaction were selected to determine the gene expression levels between the control and IC_50_ EEP groups using RT–PCR assay. The primers were designed through NCBI’s free online primer design platform, which is given in Supplementary Table S2. The internal reference gene was β-actin.

### 4.5. Xenograft Tumor Nude Mice

BALB/C male nude mice of SPF grade, 5 weeks old, weighing 18–20 g (purchased from Shanghai Jihui Experimental Animal Breeding Co., Ltd., Shanghai, China) were experimental animals. The A431 cell suspension (1 × 10^7^ cell/mL) (0.1 mL) was injected subcutaneously into the axilla of the right forelimb of nude mice. The small nodules at the inoculation site mean the heterologous tumor model in nude mice was successful. After 1 week, the tumor sizes were 4–7 mm^3^. Then, 20 nude mice were randomly divided into 4 groups: the control, solvent, 50 mg/kg EEP, and 100 mg/kg EEP groups (according to [64]). They were intragastrically administered 0.2 mL PBS buffer solution, 10% PEG-400 solution, 50 mg/kg, and 100 mg/kg. The tumor volumes of each mouse were measured every 2 days.

These nude mice were sacrificed after the final treatment. The tumor tissue was immediately peeled off with scissors and tweezers, washed with normal saline, and fixed in 4% paraformaldehyde (Biosharp, Labgic Technology Co., Ltd., Beijing, China) for paraffin section preparation and hematoxylin-eosin staining (HE, Wuhan Servicebio Technology Co., Ltd., Wuhan, China).

### 4.6. Data Analysis

All experiments were performed in triplicate. All these data are expressed as the mean ± standard error. One-way ANOVA was used to analyze the significance of differences (*p* < 0.01: extremely statistically significant differences between treatment and control groups, *p* < 0.05: statistically significant differences). The relative gene expression was represented by the ratio of gene expression in propolis-treated cells to that of control cells. Differences in tumor volumes among groups were analyzed using repeated-measures ANOVA using Stat View 5.0.1 (SAS Institute Inc. 1992–1998, Cary, NC, USA).

The spectra obtained from label-free proteomics by LC-MS/MS were analyzed as described in our previous report [9].

The raw data files generated from Untargeted Metabolomics by UHPLC-MS/MS were processed using the Compound Discoverer 3.1 (CD3.1, ThermoFisher) to perform peak alignment, peak picking, and quantitation for each metabolite, whose main parameters were set as follows: retention time tolerance, 0.2 min; actual mass tolerance, 5 ppm; signal intensity tolerance,30%; signal/noise ratio, 3; and minimum intensity.

## 5. Conclusions

A431 cancer cells can be inhibited by poplar propolis via the main pathways enriched DEPs were ECM-receptor interaction, amoebiasis, cell adhesion molecules (CAMs), nonalcoholic fatty liver disease (NAFLD) related pathway, retrograde endocannabinoid signaling, and other pathways. The inhibition effect was also found in a xenograft tumor for nude mice. Poplar propolis has the potential to be a new treatment strategy for CSCC patients.

## Figures and Tables

**Table 1:** Chemical components in ethanol extract of propolis (EEP) (full matched mzCloud).

ID	Name	Formula	Molecular Weight	Retention Time (min)	*m/z*	Relative Quantitative Value	Polarity Mode
1	1,5,8-Trihydroxy-9-oxo-9H-xanthen-3-yl β-D-glucopyranoside	C_19_H_18_O_11_	422.08375	1.31	421.07648	39,349,735.26	negative
2	D-ribose 5-phosphate	C_5_H_11_O_8_P	230.01908	1.311	229.0118	43,826,332.25	negative
3	D-Mannose 6-phosphate	C_6_H_13_O_9_P	260.02992	1.318	259.02264	349,455,529.4	negative
4	Galacturonic acid	C_6_H_10_O_7_	194.0423	1.329	193.03502	8,590,476,535	negative
5	N-Acetyl-α-D-glucosamine 1-phosphate	C_8_H_16_NO_9_P	301.05628	1.334	300.04898	31,064,500.87	negative
6	D-Saccharic acid	C_6_H_10_O_8_	210.0372	1.347	209.02992	512,353,580.7	negative
7	Gluconic acid	C_6_H_12_O_7_	196.05746	1.358	195.05019	6,478,000,924	negative
8	UDP-N-acetylglucosamine	C_17_H_27_N_3_O_17_P_2_	607.08204	1.367	606.07477	75,450,606.66	negative
9	D-(−)-Fructose	C_6_H_12_O_6_	180.06258	1.375	179.05521	2,010,181,033	negative
10	N-Acetylneuraminic acid	C_11_H_19_NO_9_	309.10545	1.383	308.09818	67,763,775.54	negative
11	L-(+)-Tartaric acid	C_4_H_6_O_6_	150.01571	1.405	149.00844	762,277,603.7	negative
12	Glucuronic acid-3,6-lactone	C_6_H_8_O_6_	176.03153	1.419	175.02422	433,003,996.6	negative
13	Sucrose	C_12_H_22_O_11_	342.11616	1.437	341.10873	16,004,974,387	negative
14	D-Raffinose	C_18_H_32_O_16_	504.16905	1.439	503.16208	1,558,022,103	negative
15	δ-Gluconic acid δ-lactone	C_6_H_10_O_6_	178.04704	1.563	177.03976	165,652,129.9	negative
16	Uric acid	C_5_H_4_N_4_O_3_	168.02751	1.886	167.02023	4,413,870,356	negative
17	D-α-Hydroxyglutaric acid	C_5_H_8_O_5_	148.03653	2.092	147.02925	149,585,529.5	negative
18	Xanthine	C_5_H_4_N_4_O_2_	152.03277	2.181	151.02548	337,332,340.5	negative
19	Uridine	C_9_H_12_N_2_O_6_	244.0697	2.415	243.06268	1,652,833,848	negative
20	N-Acetyl-DL-glutamic acid	C_7_H_11_NO_5_	189.06346	2.463	188.05618	53,573,049.69	negative
21	Ascorbic acid	C_6_H_8_O_6_	176.03149	2.814	175.02422	205,503,907.6	negative
22	2,4-Dihydroxybenzoic acid	C_7_H_6_O_4_	154.02601	3.36	153.01874	92,941,295.62	negative
23	N-(4-chlorophenyl)-N′-cyclohexylthiourea	C_13_H_17_C_l_N_2_S	268.08092	3.659	267.07364	271,608,391.5	negative
24	2-Amino-3-(4-hydroxy-3-methoxyphenyl)propanoic acid	C_10_H_13_NO_4_	211.08417	4.223	210.07693	40,438,679.16	negative
25	Xanthosine	C_10_H_12_N_4_O_6_	284.07619	4.747	283.06891	25,698,102.52	negative
26	Methylsuccinic acid	C_5_H_8_O_4_	132.04211	4.789	131.03484	147,365,715.6	negative
27	Gallic acid	C_7_H_6_O_5_	170.0212	4.884	169.01393	542,974,125	negative
28	Thymidine	C_10_H_14_N_2_O_5_	242.09074	4.895	241.08331	88,919,605.24	negative
29	3,4,5-trihydroxycyclohex-1-ene-1-carboxylic acid	C_7_H_10_O_5_	174.0523	4.907	173.04523	49,978,794.34	negative
30	1-(2,4-dihydroxyphenyl)-2-(3,5-dimethoxyphenyl)propan-1-one	C_17_H_18_O_5_	302.11583	4.93	301.10855	43,916,324.75	negative
31	5-Sulfosalicylic acid	C_7_H_6_O_6_S	217.98848	4.937	216.9812	33,337,560.45	negative
32	3′,4′-Dihydroxyphenylacetone	C_9_H_10_O_3_	166.0626	4.938	165.05533	255,487,284.2	negative
33	Porphobilinogen	C_10_H_14_N_2_O_4_	226.09543	4.963	225.08815	20,599,303.66	negative
34	D-(−)-Quinic acid	C_7_H_12_O_6_	192.06308	5.067	191.0558	687,769,173.6	negative
35	2,3-Dihydroxybenzoic acid	C_7_H_6_O_4_	154.02606	5.072	153.01874	1,430,101,121	negative
36	3-Hydroxy-3-methylglutaric acid	C_6_H_10_O_5_	162.05219	5.136	161.04492	1,232,391,129	negative
37	Vanillyl alcohol	C_8_H_10_O_3_	154.0624	5.155	153.05511	206,029,853	negative
38	L-Tyrosine methyl ester	C_10_H_13_NO_3_	195.08926	5.159	194.08197	216,011,260.4	negative
39	Phenylglyoxylic acid	C_8_H_6_O_3_	150.03111	5.166	149.02383	154,372,170.5	negative
40	Esculin	C_15_H_16_O_9_	340.08006	5.172	339.07278	51,031,924.39	negative
41	2-Isopropylmalic acid	C_7_H_12_O_5_	176.06807	5.441	175.06079	310,134,000.4	negative
42	4-((5-(4-Nitrophenyl)oxazol-2-yl)amino)benzonitrile	C_16_H_10_N_4_O_3_	306.07402	5.445	305.06674	343,517,763.8	negative
43	Miquelianin	C_21_H_18_O_13_	478.07498	5.464	477.06772	28,072,478.49	negative
44	Quercetin-3β-D-glucoside	C_21_H_20_O_12_	464.09608	5.483	463.08881	197,183,482.7	negative
45	3-Coumaric acid	C_9_H_8_O_3_	164.04692	5.521	163.03963	53,776,573,353	negative
46	Hematoxylin	C_16_H_14_O_6_	302.07832	5.539	301.07104	3,032,439,830	negative
47	Gentisic acid	C_7_H_6_O_4_	154.02598	5.542	153.01869	1,126,817,012	negative
48	Agnuside	C_22_H_26_O_11_	466.14775	5.545	465.14047	78,804,038.52	negative
49	Myricetin	C_15_H_10_O_8_	318.03764	5.554	317.03036	252,777,241.4	negative
50	Caffeic acid	C_9_H_8_O_4_	180.04162	5.562	179.03435	16,033,774,255	negative
51	3-(4-Hydroxyphenyl)propionic acid	C_9_H_10_O_3_	166.06227	5.583	165.05499	511,602,130.2	negative
52	Suberic acid	C_8_H_14_O_4_	174.08878	5.617	173.0815	805,556,199.4	negative
53	trans-Cinnamic acid	C_9_H_8_O_2_	148.05183	5.623	147.04456	299,470,310.6	negative
54	11-Dehydro thromboxane B2	C_20_H_32_O_6_	368.22037	5.64	367.21292	3,259,236,011	negative
55	Citrinin	C_13_H_14_O_5_	250.08414	5.709	249.07686	234,803,880.5	negative
56	Isorhapontigenin	C_15_H_14_O_4_	258.08848	5.722	257.08121	81,038,331.56	negative
57	DL-4-Hydroxyphenyllactic acid	C_9_H_10_O_4_	182.05747	5.728	181.05019	365,233,039.2	negative
58	3,8,9-trihydroxy-10-propyl-3,4,5,8,9,10-hexahydro-2H-oxecin-2-one	C_12_H_20_O_5_	244.1311	5.745	243.12383	213,168,051.7	negative
59	5,7-Dihydroxy-2-(3-hydroxy-4-methoxyphenyl)chroman-4-one	C_16_H_14_O_6_	302.07762	5.767	301.07034	1,212,404,197	negative
60	Quercetin	C_15_H_10_O_7_	302.04262	5.774	301.03534	26,894,567,188	negative
61	Luteolin	C_15_H_10_O_6_	286.04916	5.82	285.0419	1,272,812,181	negative
62	Naringenin	C_15_H_12_O_5_	272.06883	5.948	271.06155	2.187 × 10^11^	negative
63	β-Estradiol-17β-glucuronide	C_24_H_32_O_8_	448.21037	5.95	447.20309	389,513,176.1	negative
64	Aflatoxin G2	C_17_H_14_O_7_	330.07296	5.965	329.06567	877,430,305.3	negative
65	Monobutyl phthalate	C_12_H_14_O_4_	222.08911	6.124	221.08183	4,535,327,931	negative
66	Mycophenolic acid	C_17_H_20_O_6_	320.12593	6.188	319.11865	56,348,359.96	negative
67	Salvinorin B	C_21_H_26_O_7_	390.16923	6.221	389.16232	35,236,148.01	negative
68	Dodecanedioic acid	C_12_H_22_O_4_	230.1519	6.424	229.14462	25,219,452.38	negative
69	Aldosterone	C_21_H_28_O_5_	360.19396	6.538	359.18668	155,818,216.9	negative
70	Corchorifatty acid F	C_18_H_32_O_5_	328.22511	6.568	327.21783	1,609,573,692	negative
71	N2-(4-iodophenyl)-1,3,5-triazine-2,4-diamine	C_9_H_8_IN_5_	312.98237	6.589	311.9751	50,086,503.07	negative
72	2,3-Dinor-8-epi-prostaglandin F2α	C_18_H_30_O_5_	326.20988	6.609	325.20261	74,071,657.03	negative
73	Trolox	C_14_H_18_O_4_	250.12082	6.639	499.23444	12,254,171,786	negative
74	Genistein	C_15_H_10_O_5_	270.05308	6.658	269.04581	53,946,618,740	negative
75	(±)9(10)-DiHOME	C_18_H_34_O_4_	314.24586	6.658	313.23859	1,801,802,573	negative
76	[1,1′-biphenyl]-2,2′-dicarboxylic acid	C_14_H_10_O_4_	242.05804	6.699	241.05077	363,776,879.9	negative
77	Glycocholic acid	C_26_H_43_NO_6_	465.3098	6.753	464.30252	31,784,139.83	negative
78	Glycitein	C_16_H_12_O_5_	284.06795	6.758	283.06067	12,479,803,653	negative
79	Gibberellin A4	C_19_H_24_O_5_	332.16264	6.803	331.15536	935,893,979.2	negative
80	4-(octyloxy)benzoic acid	C_15_H_22_O_3_	250.15718	6.852	249.1499	9,593,885.513	negative
81	(±)-Abscisic acid	C_15_H_20_O_4_	264.13643	6.963	263.12915	2,145,442,724	negative
82	Tetradecanedioic acid	C_14_H_26_O_4_	258.18339	7.01	257.17612	24,368,922.87	negative
83	2-Hydroxymyristic acid	C_14_H_28_O_3_	244.2034	7.098	243.19612	262,979,901.2	negative
84	13(*S*)-HOTrE	C_18_H_30_O_3_	294.21966	7.48	293.21237	113,609,783	negative
85	Pentobarbital-d5	C_11_H_13_[2]H_5_N_2_O_3_	231.16231	7.484	230.15503	4,925,637.941	negative
86	15-keto Prostaglandin E1	C_20_H_32_O_5_	352.22508	7.857	351.21777	6,575,446.425	negative
87	16-Hydroxyhexadecanoic acid	C_16_H_32_O_3_	272.23472	7.871	271.22745	609,305,517.6	negative
88	Protoporphyrin IX	C_34_H_34_N_4_O_4_	562.25709	8.059	561.24982	7,107,790.257	negative
89	18-β-Glycyrrhetinic acid	C_30_H_46_O_4_	470.3397	8.84	469.33243	11,661,934.32	negative
90	Arachidic Acid	C_20_H_40_O_2_	312.30324	9.988	311.29596	11,584,703.86	negative
91	Docosanoic Acid	C_22_H_44_O_2_	340.33436	10.655	339.32709	5,918,139.419	negative
92	11(*Z*),14(*Z*)-Eicosadienoic Acid	C_20_H_36_O_2_	308.2715	10.673	307.26422	8,254,456.223	negative
93	Ursolic acid	C_30_H_48_O_3_	456.36055	10.725	455.35327	7,204,655.792	negative
94	Lignoceric Acid	C_24_H_48_O_2_	368.36568	10.905	367.3584	20,009,501.76	negative
95	13Z,16Z-Docosadienoic Acid	C_22_H_40_O_2_	336.30278	11.625	335.2955	41,592,258.77	negative
96	Stearic acid	C_18_H_36_O_2_	284.27189	11.678	283.26462	21,499,099.39	negative
97	N-[2-chloro-6-(trifluoromethoxy)phenyl]-2,2-dimethylpropanamide	C_12_H_13_ClF_3_NO_2_	295.05873	1.268	296.06601	165,911,372.3	positive
98	Choline	C_5_H_13_NO	103.09958	1.274	104.10686	3,351,190,589	positive
99	Glucose 1-phosphate	C_6_H_13_O_9_P	260.02959	1.332	261.03687	467,937,507.5	positive
100	Muramic acid	C_9_H_17_NO_7_	251.10009	1.337	252.10736	1,505,589,223	positive
101	Betaine	C_5_H_11_NO_2_	117.07897	1.342	118.08624	3,172,931,864	positive
102	4-Acetamidobutanoic acid	C_6_H_11_NO_3_	145.0737	1.379	146.08098	225,826,130.1	positive
103	Acetylcholine	C_7_H_15_NO_2_	145.11022	1.437	146.11749	3,685,256,761	positive
104	1-Aminocyclohexanecarboxylic acid	C_7_H_13_NO_2_	143.09483	1.438	144.10211	337,928,527.3	positive
105	Pipecolic acid	C_6_H_11_NO_2_	129.07901	1.495	130.08629	196,772,335.8	positive
106	N-Acetylhistamine	C_7_H_11_N_3_O	153.09026	1.76	154.09753	103,842,799.2	positive
107	4-Guanidinobutyric acid	C_5_H_11_N_3_O_2_	145.08527	1.843	146.09254	321,810,960.4	positive
108	Nicotinic acid	C_6_H_5_NO_2_	123.03225	1.945	124.03953	839,395,599.9	positive
109	Nicotinamide	C_6_H_6_N_2_O	122.0483	2.071	123.05557	569,720,151.5	positive
110	6-Hydroxynicotinic acid	C_6_H_5_NO_3_	139.02706	2.112	140.03433	1,036,882,714	positive
111	Pyrrole-2-carboxylic acid	C_5_H_5_NO_2_	111.03238	2.12	112.03966	479,797,100.7	positive
112	L-Pyroglutamic acid	C_5_H_7_NO_3_	129.0428	2.175	130.05005	620,786,771.7	positive
113	3-isopropoxy-4-morpholinocyclobut-3-ene-1,2-dione	C_11_H_15_NO_4_	225.1004	2.395	226.10768	67,026,578.08	positive
114	Uracil	C_4_H_4_N_2_O_2_	112.02752	2.441	113.0348	870,107,158.9	positive
115	Adenine	C_5_H_5_N_5_	135.05466	3.527	136.06194	330,830,026.4	positive
116	Adenosine	C_10_H_13_N_5_O_4_	267.09682	3.529	268.1041	1,427,095,688	positive
117	Inosine	C_10_H_12_N_4_O_5_	268.08094	3.964	269.08823	257,548,392.1	positive
118	Hypoxanthine	C_5_H_4_N_4_O	136.03859	3.965	137.04587	887,426,643.9	positive
119	Diethyl phosphate	C_4_H_11_O_4_P	154.03959	3.974	155.04686	399,273,696.2	positive
120	DL-Stachydrine	C_7_H_13_NO_2_	143.09484	4.175	144.10211	1,831,709,107	positive
121	D(−)-Amygdalin	C_20_H_27_NO_11_	457.1595	4.838	458.16678	209,671,201.6	positive
122	N-[3-(2-methyl-4-pyrimidinyl)phenyl]-1,3-benzothiazole-2-carboxamide	C_19_H_14_N_4_OS	346.08824	4.856	347.09552	24,561,559.93	positive
123	1-methyl-2-oxo-1,2-dihydroquinolin-4-yl N,N-dimethylcarbamate	C_13_H_14_N_2_O_3_	246.10079	4.876	247.10806	37,336,553.33	positive
124	N-[1-(4-methoxy-2-oxo-2H-pyran-6-yl)-2-methylbutyl]acetamide	C_13_H_19_NO_4_	253.1315	4.899	254.13878	63,994,227.66	positive
125	2-(tert-butyl)-6,7-dimethoxy-4H-3,1-benzoxazin-4-one	C_14_H_17_NO_4_	263.11608	4.901	264.12335	185,237,827.6	positive
126	L-Dopa	C_9_H_11_NO_4_	197.06899	4.906	198.07626	140,151,015.2	positive
127	2-(2-acetyl-3,5-dihydroxyphenyl)acetic acid	C_10_H_10_O_5_	210.053	4.926	211.06032	98,428,683	positive
128	1-(3,4-dimethoxyphenyl)ethan-1-one oxime	C_10_H_13_NO_3_	195.08971	4.933	196.09698	614,007,207	positive
129	4-Hydroxybenzaldehyde	C_7_H_6_O_2_	122.03695	4.95	123.0442	432,952,790.1	positive
130	Retrorsine	C_18_H_25_NO_6_	351.16835	4.971	352.17566	454,873,409.5	positive
131	N-(5-methylisoxazol-3-yl)-N’-[4-(trifluoromethyl)-3-pyridyl]urea	C_11_H_9_F_3_N_4_O_2_	286.06688	4.99	287.07416	26,800,545.08	positive
132	2-[(carboxymethyl)(methyl)amino]-5-methoxybenzoic acid	C_11_H_13_NO_5_	239.07959	5.014	240.08687	364,769,903.8	positive
133	5-[(2-hydroxybenzylidene)amino]-2-(2-methoxyethoxy)benzoic acid	C_17_H_17_NO_5_	315.11092	5.047	316.11819	71,187,584.76	positive
134	2-(3,4-dihydroxyphenyl)acetamide	C_8_H_9_NO_3_	167.05841	5.09	168.06569	160,810,937.1	positive
135	3-Methoxybenzaldehyde	C_8_H_8_O_2_	136.05248	5.141	137.05975	1,453,566,975	positive
136	cis,cis-Muconic acid	C_6_H_6_O_4_	142.02675	5.227	143.03403	370,439,328.5	positive
137	Xanthurenic acid	C_10_H_7_NO_4_	205.03758	5.245	206.04486	142,669,582.9	positive
138	3-Methylcrotonylglycine	C_7_H_11_NO_3_	157.074	5.255	158.08128	1,115,733,525	positive
139	3-hydroxy-3,4-bis[(4-hydroxy-3-methoxyphenyl)methyl]oxolan-2-one	C_20_H_22_O_7_	374.13646	5.256	375.14374	53,726,456.67	positive
140	5,6-dimethyl-4-oxo-4H-pyran-2-carboxylic acid	C_8_H_8_O_4_	168.0423	5.292	169.04958	265,935,155.1	positive
141	8-Hydroxyquinoline	C_9_H_7_NO	145.05283	5.294	146.0601	260,410,244.6	positive
142	Kynurenic acid	C_10_H_7_NO_3_	189.04259	5.32	190.04987	1,905,007,525	positive
143	Safrole	C_10_H_10_O_2_	162.06841	5.32	163.07619	212,114,032.4	positive
144	Bergapten	C_12_H_8_O_4_	216.04233	5.336	217.04961	134,452,483.5	positive
145	Isoferulic acid	C_10_H_10_O_4_	194.05797	5.342	195.06525	2,726,827,967	positive
146	1-(4-butylphenyl)-3-(dimethylamino)propan-1-one hydrochloride	C_15_H_23_NO	233.17806	5.354	234.18533	95,286,786.45	positive
147	2-Phenylglycine	C_8_H_9_NO_2_	151.06335	5.394	152.07062	248,042,836.8	positive
148	N1-(2,3-dihydro-1,4-benzodioxin-6-yl)acetamide	C_10_H_11_NO_3_	193.07393	5.394	194.08121	470,764,198.3	positive
149	Isohomovanillic acid	C_9_H_10_O_4_	182.05789	5.476	183.06517	632,371,956.6	positive
150	Resveratrol	C_14_H_12_O_3_	228.0786	5.495	229.08588	127,433,383.6	positive
151	Vanillin	C_8_H_8_O_3_	152.04734	5.5	153.05461	7,449,671,173	positive
152	2-(2-hydroxy-3-methylbutanamido)-4-methylpentanoic acid	C_11_H_21_NO_4_	231.14748	5.507	232.15465	16,473,889.02	positive
153	4-Coumaric acid	C_9_H_8_O_3_	164.04727	5.509	165.05449	10,555,435,607	positive
154	7-hydroxy-3-phenyl-4H-chromen-4-one	C_15_H_10_O_3_	238.06268	5.518	239.06996	48,729,023.77	positive
155	Apocynin	C_9_H_10_O_3_	166.06305	5.52	167.07033	570,626,404.7	positive
156	4-Phenylbutyric acid	C_10_H_12_O_2_	164.08381	5.539	165.09109	205,439,194.1	positive
157	Ferulic acid	C_10_H_10_O_4_	194.05799	5.545	195.06525	75,400,334,073	positive
158	3-benzyl-4-hydroxy-5-(4-hydroxyphenyl)-2,5-dihydrofuran-2-one	C_17_H_14_O_4_	282.08907	5.564	283.09634	91,049,837.1	positive
159	Trifolin	C_21_H_20_O_11_	448.10082	5.574	449.10809	207,852,942.9	positive
160	2-[5-(2-hydroxypropyl)oxolan-2-yl]propanoic acid	C_10_H_18_O_4_	202.1205	5.618	203.12778	89,488,627.25	positive
161	Isoeugenyl acetate	C_12_H_14_O_3_	206.09445	5.678	207.10175	107,711,395.8	positive
162	(5E)-7-methylidene-10-oxo-4-(propan-2-yl)undec-5-enoic acid	C_15_H_24_O_3_	252.17266	5.712	253.17993	1,402,040,442	positive
163	(2S)-2-(2-hydroxypropan-2-yl)-2H,3H,7H-furo[3,2-g]chromen-7-one	C_14_H_14_O_4_	246.0892	5.717	247.09648	1,605,550,194	positive
164	3,4-Dimethoxycinnamic acid	C_11_H_12_O_4_	208.0736	5.72	209.08086	63,552,367,246	positive
165	Biochanin A	C_16_H_12_O_5_	284.06853	5.722	285.07581	5,527,495,198	positive
166	4-Methoxycinnamaldehyde	C_10_H_10_O_2_	162.06807	5.722	163.07535	1,922,721,932	positive
167	Piceatannol	C_14_H_12_O_4_	244.07353	5.735	245.08081	268,412,733.9	positive
168	Hesperetin	C_16_H_14_O_6_	302.07887	5.748	303.08615	232,819,296.3	positive
169	7-hydroxy-3-(4-methoxyphenyl)-4H-chromen-4-one	C_16_H_12_O_4_	268.07356	5.759	269.08084	1,376,762,031	positive
170	Galangin	C_15_H_10_O_5_	270.05278	5.766	271.06006	18,105,451,784	positive
171	(2R)-2-[(2R,5S)-5-[(2S)-2-hydroxybutyl]oxolan-2-yl]propanoic acid	C_11_H_20_O_4_	216.13623	5.791	217.14351	217,544,557.8	positive
172	(1E,4Z,6E)-5-hydroxy-1,7-bis(4-hydroxyphenyl)hepta-1,4,6-trien-3-one	C_19_H_16_O_4_	308.10472	5.809	309.112	46,531,520.91	positive
173	3-hydroxy-4-methoxy-9H-xanthen-9-one	C_14_H_10_O_4_	242.05791	5.827	243.06519	295,235,024	positive
174	Daidzin	C_21_H_20_O_9_	416.11073	5.835	417.11801	638,726,156.6	positive
175	4,7-dimethoxy-1H-phenalen-1-one	C_15_H_12_O_3_	240.07863	5.844	241.08591	3,312,421,173	positive
176	Naringeninchalcone	C_15_H_12_O_5_	272.06831	5.952	273.07559	18,351,009,032	positive
177	12-Oxo phytodienoic acid	C_18_H_28_O_3_	292.20342	5.963	293.21069	2,505,657,284	positive
178	Kaempferol	C_15_H_10_O_6_	286.04823	5.967	287.05551	9,006,992,981	positive
179	2,4-Dimethylbenzaldehyde	C_9_H_10_O	134.0734	5.985	117.07014	1,223,537,063	positive
180	4-Methoxycinnamic acid	C_10_H_10_O_3_	178.06309	6.002	179.0703	6,932,438,506	positive
181	(+)-ar-Turmerone	C_15_H_20_O	216.15139	6.016	217.15866	1,027,389,042	positive
182	Apigenin	C_15_H_10_O_5_	270.05278	6.024	271.06006	14,095,816,138	positive
183	6-Pentyl-2H-pyran-2-one	C_10_H_14_O_2_	166.09939	6.049	167.10667	903,441,178.6	positive
184	Methyl cinnamate	C_10_H_10_O_2_	162.06808	6.071	163.07535	455,777,480.5	positive
185	4-Phenyl-3-buten-2-one	C_10_H_10_O	146.07324	6.076	147.08052	483,099,106.7	positive
186	5-(2,5-dihydroxyhexyl)oxolan-2-one	C_10_H_18_O_4_	202.12056	6.094	203.12785	98,842,367.03	positive
187	Cardamomin	C_16_H_14_O_4_	270.08916	6.121	271.09644	17,850,179,874	positive
188	Citral	C_10_H_16_O	152.12017	6.184	153.12744	3,164,365,769	positive
189	Formononetin	C_16_H_12_O_4_	268.07366	6.228	269.08093	5,354,174,356	positive
190	Rhamnetin	C_16_H_12_O_7_	316.05788	6.28	317.06516	5,100,142,040	positive
191	3-Methoxyflavone	C_16_H_12_O_3_	252.07872	6.313	253.086	477,044,431	positive
192	trans-Cinnamaldehyde	C_9_H_8_O	132.05778	6.316	133.06496	3,462,748,945	positive
193	5-hydroxy-6,7-dimethoxy-2-phenyl-4H-chromen-4-one	C_17_H_14_O_5_	298.08406	6.338	299.09134	474,558,537.9	positive
194	4-Hydroxybenzophenone	C_13_H_10_O_2_	198.06813	6.349	199.07541	858,164,155.1	positive
195	Sakuranetin	C_16_H_14_O_5_	286.08367	6.398	287.09094	10,055,131,651	positive
196	Veratrole	C_8_H_10_O_2_	138.06813	6.414	139.07541	1,285,393,336	positive
197	Pinocembrin	C_15_H_12_O_4_	256.07356	6.416	257.08084	19,143,801,362	positive
198	Nootkatone	C_15_H_22_O	218.16721	6.436	219.17448	7,475,410,243	positive
199	1-oxo-2,3-dihydro-1H-inden-4-yl benzoate	C_16_H_12_O_3_	252.07872	6.481	253.086	86,170,158.6	positive
200	3,14-dihydro-15-keto-tetranor Prostaglandin E2	C_16_H_26_O_5_	298.17794	6.505	299.18539	363,566,952.2	positive
201	Carvone	C_10_H_14_O	150.10456	6.555	151.11183	1,494,122,211	positive
202	Chrysin	C_15_H_10_O_4_	254.05795	6.577	255.06523	53,403,620,396	positive
203	Coenzyme Q2	C_19_H_26_O_4_	318.18284	6.717	319.19012	250,369,688.1	positive
204	WNK	C_21_H_30_N_6_O_5_	446.22838	6.72	447.23566	49,336,245.15	positive
205	Wogonin	C_16_H_12_O_5_	284.06853	6.735	285.07581	16,003,176,213	positive
206	1,7,8-trihydroxy-3-methyl-1,2,3,4,7,12-hexahydrotetraphen-12-one	C_19_H_18_O_4_	310.12057	6.884	311.1279	66,568,647.86	positive
207	4-methoxy-6-(prop-2-en-1-yl)-2H-1,3-benzodioxole	C_11_H_12_O_3_	192.07906	6.95	193.08633	6,455,924.178	positive
208	9-Oxo-ODE	C_18_H_30_O_3_	294.21946	7	295.22672	8,284,510,598	positive
209	(2R)-5-hydroxy-7-methoxy-2-phenyl-3,4-dihydro-2H-1-benzopyran-4-one	C_16_H_14_O_4_	270.08928	7.074	271.09656	638,934,307.1	positive
210	(−)-Caryophyllene oxide	C_15_H_24_O	220.18288	7.191	221.19016	4,440,500,049	positive
211	9-Oxo-10(E),12(E)-octadecadienoic acid	C_18_H_30_O_3_	294.21948	7.935	295.22681	10,127,470,520	positive
212	DL-Dipalmitoylphosphatidylcholine	C_40_H_80_NO_8_P	733.56401	10.387	734.57129	171,680,834.5	positive
213	Betulin	C_30_H_50_O_2_	442.38181	10.556	443.38919	131,107,308.5	positive
214	Palmitoyl sphingomyelin	C_39_H_79_N_2_O_6_P	702.56926	11.002	703.57654	741,892,482.6	positive

**Table 2:** Partial significant (*p* < 0.01) differentially expressed proteins (DEPs) between the ethanol extract of propolis (EEP) group and the control group (screened by FC > 2.0 or FC < 0.5 and *p* < 0.05).

Protein ID	Name	*p*	Regulated
O75911	Short-chain dehydrogenase/reductase 3	0.000141468	down
Q9UPY5	Cystine/glutamate transporter	0.000659187	up
Q6IBA0	NADH dehydrogenase (Ubiquinone) Fe-S protein 5, 15 kDa (NADH-coenzyme Q reductase)	0.00094246	down
A0A024RDX4	ATP-dependent (S)-NAD(P)H-hydrate dehydratase	0.001028075	up
Q9Y4K0	Lysyl oxidase homolog 2	0.001568804	up
Q03405	Urokinase plasminogen activator surface receptor	0.001635932	up
Q96JY6	PDZ and LIM domain protein 2	0.001648702	up
A0A024R084	Stromal cell-derived factor 4, isoform CRA_c	0.001660675	down
A0A0A6YYF2	HCG1811249, isoform CRA_e	0.001700981	up
B3KN79	cDNA FLJ13894 fis, clone THYRO1001671, highly similar to 59 kDa 2-5-oligoadenylate synthetase-like protein	0.002052659	down
A6NCE7	Microtubule-associated proteins 1A/1B light chain 3 beta 2	0.002064172	up
Q9BXY0	Protein MAK16 homolog	0.003155254	down
Q14139	Ubiquitin conjugation factor E4 A	0.003259227	up
A8K878	Mesencephalic astrocyte-derived neurotrophic factor	0.003402074	up
Q9NQC3	Reticulon-4	0.003468251	up
Q9Y316	Protein MEMO1	0.003479756	down
E7EPT4	NADH dehydrogenase [ubiquinone] flavoprotein 2, mitochondrial	0.003643833	down
P18827	Syndecan-1	0.004028676	up
P49821	NADH dehydrogenase [ubiquinone] flavoprotein 1, mitochondrial	0.004095327	down
A8K0B9	rRNA adenine N(6)-methyltransferase	0.004645847	down
A0A3B3ISF9	Endothelin-converting enzyme 1	0.004659358	down
Q96PU5	E3 ubiquitin-protein ligase NEDD4-like	0.004703096	down
Q9NX12	cDNA FLJ20496 fis, clone KAT08729	0.005418258	up
Q53HG1	NADH dehydrogenase [ubiquinone] 1 alpha subcomplex subunit 12 (Fragment)	0.005954737	down
Q14684	Ribosomal RNA processing protein 1 homolog B	0.006713584	down
Q04828	Aldo-keto reductase family 1 member C1	0.006731072	up
Q9GZM7	Tubulointerstitial nephritis antigen-like	0.007059725	up
P22223	Cadherin-3	0.00794582	up
O95167	NADH dehydrogenase [ubiquinone] 1 alpha subcomplex subunit 3	0.008545123	down
Q496C9	D-aminoacyl-tRNA deacylase	0.008949922	up
A8K8P8	Alpha-(1,6)-fucosyltransferase	0.00914376	down
B4DTK7	cDNA FLJ61387, highly similar to *Homo sapiens* conserved nuclear protein NHN1 (NHN1), Mrna	0.009420668	up
A0A024R1I7	Tuftelin-interacting protein 11	0.009715326	up

**Table 3:** The significantly enriched pathways (adjusted *p* < 0.05) of differentially expressed proteins.

Map Title	Adjusted *p* Value	Regulated	Description
ECM-receptor interaction	5.55 × 10^−5^	up	Laminin subunit beta-3, HCG1811249,isoform CRA_e, Laminin subunit gamma-2, Fibronectin 1, isoform CRA_n, Thrombospondin 1, isoform CRA_a, Agrin, Syndecan-1
Amoebiasis	0.000108083	up	Laminin subunit beta-3, HCG1811249, isoform CRA_e, Laminin subunit gamma-2, Fibronectin 1, isoform CRA_n, Ras-related protein Rab-5B, Serpin B6, Leukocyte elastase inhibitor
Cell adhesion molecules (CAMs)	0.000267166	up	Cadherin-3, Cadherin-1, Syndecan-1, Programmed cell death 1 ligand 1, Occludin, MHC class I antigen (Fragment), MHC class I antigen (Fragment)
Nonalcoholic fatty liver disease (NAFLD)	1.44 × 10^−11^	down	Succinate dehydrogenase [ubiquinone] flavoprotein subunit, mitochondrial,Mitochondrial NADH-ubiquinone oxidoreductase 75 kDa subunit, NADH dehydrogenase [ubiquinone] flavoprotein 1, mitochondrial, cDNA FLJ75930, highly similar to *Homo sapiens* NADH dehydrogenase (ubiquinone) 1 alpha subcomplex, 9, 39 kDa (NDUFA9), mRNA, NADH dehydrogenase [ubiquinone] flavoprotein 2, mitochondrial, NDUFA7 protein (Fragment), NADH dehydrogenase (Ubiquinone) Fe-S protein 5, 15 kDa (NADH-coenzyme Q reductase), NADH dehydrogenase [ubiquinone] 1 alpha subcomplex subunit 6, NADH dehydrogenase [ubiquinone] 1 alpha subcomplex subunit 2, NADH dehydrogenase [ubiquinone] iron-sulfur protein 4, mitochondrial,NADH dehydrogenase [ubiquinone] iron-sulfur protein 8, mitochondrial, NADH dehydrogenase [ubiquinone] 1 alpha subcomplex subunit 12 (Fragment), NADH dehydrogenase [ubiquinone] iron-sulfur protein 7, mitochondrial, NADH dehydrogenase [ubiquinone] 1 alpha subcomplex subunit 3, NADH dehydrogenase [ubiquinone] 1 beta subcomplex subunit 3, Uncharacterized protein (Fragment), Inhibitor of nuclear factor kappa-B kinase subunit beta
Retrograde endocannabinoid signaling	1.44 × 10^−11^	down	Mitochondrial NADH-ubiquinone oxidoreductase 75 kDa subunit, NADH dehydrogenase [ubiquinone] flavoprotein 1, mitochondrial, cDNA FLJ75930, highly similar to *Homo sapiens* NADH dehydrogenase (ubiquinone) 1 alpha subcomplex, 9, 39 kDa (NDUFA9), mRNA, NADH dehydrogenase [ubiquinone] flavoprotein 2, mitochondrial, NDUFA7 protein (Fragment), NADH dehydrogenase (Ubiquinone) Fe-S protein 5, 15 kDa (NADH-coenzyme Q reductase), NADH dehydrogenase [ubiquinone] 1 alpha subcomplex subunit 6, NADH dehydrogenase [ubiquinone] 1 alpha subcomplex subunit 2, NADH dehydrogenase [ubiquinone] iron-sulfur protein 4, mitochondrial, NADH dehydrogenase [ubiquinone] iron-sulfur protein 8, mitochondrial, NADH dehydrogenase [ubiquinone] 1 alpha subcomplex subunit 12 (Fragment), NADH dehydrogenase [ubiquinone] iron-sulfur protein 7, mitochondrial, NADH dehydrogenase [ubiquinone] 1 alpha subcomplex subunit 3, NADH dehydrogenase [ubiquinone] 1 beta subcomplex subunit 3, Uncharacterized protein (Fragment)
Alzheimer’s disease	7.51 × 10^−10^	down	Succinate dehydrogenase [ubiquinone] flavoprotein subunit, mitochondrial, Mitochondrial NADH-ubiquinone oxidoreductase 75 kDa subunit,NADH dehydrogenase [ubiquinone] flavoprotein 1, mitochondrial,cDNA FLJ75930, highly similar to *Homo sapiens* NADH dehydrogenase (ubiquinone) 1 alpha subcomplex, 9, 39 kDa (NDUFA9), mRNA,NADH dehydrogenase [ubiquinone] flavoprotein 2, mitochondrial,NDUFA7 protein (Fragment),NADH dehydrogenase (Ubiquinone) Fe-S protein 5, 15 kDa (NADH-coenzyme Q reductase),NEDD8-activating enzyme E1 regulatory subunit,NADH dehydrogenase [ubiquinone] 1 alpha subcomplex subunit 6,NADH dehydrogenase [ubiquinone] 1 alpha subcomplex subunit 2,NADH dehydrogenase [ubiquinone] iron-sulfur protein 4, mitochondrial,NADH dehydrogenase [ubiquinone] iron-sulfur protein 8, mitochondrial,NADH dehydrogenase [ubiquinone] 1 alpha subcomplex subunit 12 (Fragment),NADH dehydrogenase [ubiquinone] iron-sulfur protein 7, mitochondrial,NADH dehydrogenase [ubiquinone] 1 alpha subcomplex subunit 3,NADH dehydrogenase [ubiquinone] 1 beta subcomplex subunit 3,Uncharacterized protein (Fragment)
Oxidative phosphorylation	1.02 × 10^−9^	down	Succinate dehydrogenase [ubiquinone] flavoprotein subunit, mitochondrial,Mitochondrial NADH-ubiquinone oxidoreductase 75 kDa subunit,NADH dehydrogenase [ubiquinone] flavoprotein 1, mitochondrial,cDNA FLJ75930, highly similar to *Homo sapiens* NADH dehydrogenase (ubiquinone) 1 alpha subcomplex, 9, 39 kDa (NDUFA9), mRNA,NADH dehydrogenase [ubiquinone] flavoprotein 2, mitochondrial,NDUFA7 protein (Fragment),NADH dehydrogenase (Ubiquinone) Fe-S protein 5, 15 kDa (NADH-coenzyme Q reductase),NADH dehydrogenase [ubiquinone] 1 alpha subcomplex subunit 6,NADH dehydrogenase [ubiquinone] 1 alpha subcomplex subunit 2,NADH dehydrogenase [ubiquinone] iron-sulfur protein 4, mitochondrial,NADH dehydrogenase [ubiquinone] iron-sulfur protein 8, mitochondrial,NADH dehydrogenase [ubiquinone] 1 alpha subcomplex subunit 12 (Fragment),NADH dehydrogenase [ubiquinone] iron-sulfur protein 7, mitochondrial,NADH dehydrogenase [ubiquinone] 1 alpha subcomplex subunit 3,NADH dehydrogenase [ubiquinone] 1 beta subcomplex subunit 3,Uncharacterized protein (Fragment)
Parkinson’s disease	1.02 × 10^−9^	down	Succinate dehydrogenase [ubiquinone] flavoprotein subunit, mitochondrial,Mitochondrial NADH-ubiquinone oxidoreductase 75 kDa subunit,NADH dehydrogenase [ubiquinone] flavoprotein 1, mitochondrial,cDNA FLJ75930, highly similar to *Homo sapiens* NADH dehydrogenase (ubiquinone) 1 alpha subcomplex, 9, 39 kDa (NDUFA9), mRNA,NADH dehydrogenase [ubiquinone] flavoprotein 2, mitochondrial,NDUFA7 protein (Fragment),NADH dehydrogenase (Ubiquinone) Fe-S protein 5, 15 kDa (NADH-coenzyme Q reductase),NADH dehydrogenase [ubiquinone] 1 alpha subcomplex subunit 6,NADH dehydrogenase [ubiquinone] 1 alpha subcomplex subunit 2,NADH dehydrogenase [ubiquinone] iron-sulfur protein 4, mitochondrial,NADH dehydrogenase [ubiquinone] iron-sulfur protein 8, mitochondrial,NADH dehydrogenase [ubiquinone] 1 alpha subcomplex subunit 12 (Fragment),NADH dehydrogenase [ubiquinone] iron-sulfur protein 7, mitochondrial,NADH dehydrogenase [ubiquinone] 1 alpha subcomplex subunit 3,NADH dehydrogenase [ubiquinone] 1 beta subcomplex subunit 3,Uncharacterized protein (Fragment)
Huntington’s disease	3.50 × 10^−8^	down	Succinate dehydrogenase [ubiquinone] flavoprotein subunit, mitochondrial,Mitochondrial NADH-ubiquinone oxidoreductase 75 kDa subunit,NADH dehydrogenase [ubiquinone] flavoprotein 1, mitochondrial,cDNA FLJ75930, highly similar to *Homo sapiens* NADH dehydrogenase (ubiquinone) 1 alpha subcomplex, 9, 39 kDa (NDUFA9), mRNA,NADH dehydrogenase [ubiquinone] flavoprotein 2, mitochondrial,NDUFA7 protein (Fragment),NADH dehydrogenase (Ubiquinone) Fe-S protein 5, 15 kDa (NADH-coenzyme Q reductase),NADH dehydrogenase [ubiquinone] 1 alpha subcomplex subunit 6,NADH dehydrogenase [ubiquinone] 1 alpha subcomplex subunit 2,NADH dehydrogenase [ubiquinone] iron-sulfur protein 4, mitochondrial,NADH dehydrogenase [ubiquinone] iron-sulfur protein 8, mitochondrial,NADH dehydrogenase [ubiquinone] 1 alpha subcomplex subunit 12 (Fragment),NADH dehydrogenase [ubiquinone] iron-sulfur protein 7, mitochondrial,NADH dehydrogenase [ubiquinone] 1 alpha subcomplex subunit 3,NADH dehydrogenase [ubiquinone] 1 beta subcomplex subunit 3,Uncharacterized protein (Fragment)
Metabolic pathways	5.47 × 10^−7^	down	Succinate dehydrogenase [ubiquinone] flavoprotein subunit, mitochondrial,Mitochondrial NADH-ubiquinone oxidoreductase 75 kDa subunit,NADH dehydrogenase [ubiquinone] flavoprotein 1, mitochondrial,cDNA FLJ75930, highly similar to *Homo sapiens* NADH dehydrogenase (ubiquinone) 1 alpha subcomplex, 9, 39 kDa (NDUFA9), mRNA,NADH dehydrogenase [ubiquinone] flavoprotein 2, mitochondrial,Drug-sensitive protein 1,Alpha-(1,6)-fucosyltransferase,Short-chain dehydrogenase/reductase 3,NDUFA7 protein (Fragment),NADH dehydrogenase (Ubiquinone) Fe-S protein 5, 15 kDa (NADH-coenzyme Q reductase),NADH dehydrogenase [ubiquinone] 1 alpha subcomplex subunit 6,Ferrochelatase, mitochondrial,NADH dehydrogenase [ubiquinone] 1 alpha subcomplex subunit 2,NADH dehydrogenase [ubiquinone] iron-sulfur protein 4, mitochondrial,NADH dehydrogenase [ubiquinone] iron-sulfur protein 8, mitochondrial,NADH dehydrogenase [ubiquinone] 1 alpha subcomplex subunit 12 (Fragment),NADH dehydrogenase [ubiquinone] iron-sulfur protein 7, mitochondrial,2-oxoisovalerate dehydrogenase subunit alpha,N-acetylgalactosaminyltransferase 7,Amidophosphoribosyltransferase,NADH dehydrogenase [ubiquinone] 1 alpha subcomplex subunit 3,NADH dehydrogenase [ubiquinone] 1 beta subcomplex subunit 3,Uncharacterized protein (Fragment),DNA-directed RNA polymerase I subunit RPA2,Biliverdin reductase A,Dol-P-Man:Man(5)GlcNAc(2)-PP-Dol alpha-1,3-mannosyltransferase,Phosphoribosylformylglycinamidine synthase (FGAR amidotransferase), isoform CRA_b, Dihydropyrimidinase, UDP-Gal:betaGlcNAc beta 1,4-galactosyltransferase, polypeptide 4
Glycosaminoglycan biosynthesis—keratan sulfate	0.025255288	down	Alpha-(1,6)-fucosyltransferase, UDP-Gal:betaGlcNAc beta 1,4-galactosyltransferase, polypeptide 4

**Table 4:** Volume of A431 cell xenograft tumors in nude mice (mm^3^, *n* = 5).

Time (Days)	Control	Solvent Control	50 mg/kg EEP	100 mg/kg EEP
0	106.9 ± 21.64	108.06 ± 16.09	127.03 ± 31.07	153 ± 32.99
3	297.37 ± 43.17	223.74 ± 40.86	299.84 ± 68.85	267.42 ± 25.71
6	472.45 ± 64.62	368.35 ± 51.45	505.06 ± 112.64	424.31 ± 58.3
9	708.31 ± 74.26	696.13 ± 145.68	765.78 ± 169.3	530.67 ± 57.45
12	919.71 ± 56.47	1118.4 ± 239.05	1001.22 ± 202.33	771.04 ± 79.93 *

* Mean differences compared with the control group (*p* < 0.05).

## Data Availability

Data are contained within this article and supplementary materials.

## Supplementary Materials

The supporting information can be downloaded at https://www.mdpi.com/article/10.3390/ijms242316753/s1.
