# Supplementary material for: Antitumor Effect of Poplar Propolis on Human Cutaneous Squamous Cell Carcinoma A431 Cells
Source: Int J Mol Sci. 2023 Nov 25;24(23):16753. doi: 10.3390/ijms242316753 (PMC10706191; doi:10.3390/ijms242316753)
Supplement: Supplementary file 1 [file ijms-24-16753-s001.zip › ijms-2673827-supplementary.pdf]

Supplemental files

Table S1 The cycle threshold (ct) data of selected genes.

Gene	Control	Control	Control	Treatment	Treatment	Treatment
Actin	16.13	16.35	16.43	17.97	18.03	17.86
FN1	18.36	17.73	18.07	20.04	19.18	20.33
LAMC1	21.00	20.30	20.59	20.80	20.84	20.15
SDC1	35.49	28.28	27.27	27.73	27.01	27.59
THBS1	25.99	25.36	25.01	26.31	25.92	26.50
NDUFA2	24.47	24.42	24.33	25.05	23.82	24.38
NDUFS1	26.31	26.09	26.10	26.43	26.20	26.19
NDUFV1	28.99	29.06	29.42	28.84	28.94	29.09
SDHA	21.55	21.75	22.15	22.58	23.00	22.89

Table S2 Primer sequences for PCR amplification

**Primer name**	**Primer sequences（5'to3'）**
β-actin F	GATCATTGCTCCTCCTGAGC
β-actin R	ACTCCTGCTTGCTGATCCAC
LAMC1 F	GCATCTGCATCTCCTACCCC
LAMC1 R	CGGATGGCTCAGTGTCCTAC
SDC1 F	GAGCTGAAAGGCCGGGAAC
SDC1 R	CTACTGCCGGATTCCTCTCC
THBS1 F	ACAGTAAGTCATAGCAACATTCACA
THBS1 R	GGGGCCCTTGACTTAGGGAT
NDUFS1 F	AAAGGATGTGGGAAGGTGTGAT
NDUFS1 R	GCACGCTTCCCCTCTAAAAA
NDUFV1 F	TGAAGGTGACAGCGTGAGGT
NDUFV1 R	TTCTTGGGTGCTGTCCGC
SDHA F	TGCCATCCACTACATGACGG
SDHA R	GCTCTGTCCACCAAATGCAC
